# The Editor's Letter-Box

**Published:** 1917-03-31

**Authors:** Helen Wilson, Charles J. Tarring, Alison Neilans

**Affiliations:** Chairman.; Secretary


### THE EDITOR'S LETTER-BOX.

### The Criminal Law Amendment Bill.

Alison Neilans, *Secretary.*

The Association for Moral and Social Hygiene,

19 Tothill Street, Westminster, S.W. 1,

March 19, 1917.

### Copy of Letter Signed by Dr. Helen Wilson and the Chairman and Secretary of the Association for Moral and Social Hygiene.

CRIMINAL LAW AMENDMENT ACT.

Sir,—We are desired by the executive committee of the Association for Moral and Social Hygiene to forward the following comments upon the Criminal Law Amendment Bill.

While generally approving most of the clauses, with the exception of Clauses 2 and 6, we should welcome the amendment of Clause 1 (which deals with the protection of girls under sixteen from acts of indecency) to include the protection of boys.

We are opposed to Clauses 2 and 6, and urge their deletion. *From first to last the Bill makes no attempt to make the communication of venereal disease a punishable offence.* Clause 2 prohibits sexual intercourse or even soliciting to sexual intercourse, under certain conditions. It does not profess to deal with other ways in which this disease may be communicated.

We take the strongest exception to sub-sections 3 and 4 of Clause 2. Sub-section 3 gives power to a magistrate or judge to subject a person to a degrading physical examination, which, in the case of women, amounts to a positive outrage. Sub-section 4 is in effect merely an indirect method of making a woman liable to two years' imprisonment, for women constitute the vast majority of the persons convicted of the offences mentioned in the Schedule. *The particular injustice of Sub-sections 3 and 4 lies in the fact that this heavy sentence and forced medical examination can be imposed upon women without any evidence, other than police evidence, being given at any stage, that any person was infected, solicited, or molested by the women accused.*

We suggest the following substitute for Clause 2 as being simple and effective and free from contentious elements :—

"Any person who knowingly, or by culpable negligence, communicates venereal disease to any other person shall be deemed to be guilty of causing grievous bodily harm, and shall be liable on conviction to the penalties mentioned in Clause 2 of the Bill."

Clause 6 merely increases the penalty on a second conviction for solicitation. The whole law on solicitation is hopelessly confused and indefinite, and in practice is only applied to women. It should therefore be entirely swept away, and for it should be substituted a law which prohibits molestation or annoyance in the streets by any person or persons, no convictions to follow except on the evidence of the person so molested or annoyed. It is only when solicitation amounts to annoyance that it should be treated as an offence. If it does not cause annoyance the person solicited has no cause of complaint. If this be considered too sweeping a change to be made in war-time, we do at least demand that the inequality between men and women shall not be increased by imposing further penalties for women only.—Yours faithfully,

Helen Wilson
Charles J. Tarring, *Chairman.*
Alison Neilans, *Secretary.*

[Dr. Helen Wilson's defence is not much to the purpose. She and her coadjutors wish the first clause of the Bill to give protection to boys as well as to girls under the age of sixteen. This does not meet the case we put, that when indecency is committed with a girl the girl is often as guilty, and often more guilty, than her partner. To say, as this new pronouncement does, that from first to last the Bill makes no attempt to make the communication of venereal disease a punishable offence is not only untrue but grossly untrue. If inflicting the punishment of two years' hard labour on a person who has sexual intercourse while suffering from venereal disease in a communicable form is not an attempt to make the communication of these diseases a punishable offence, then no measure can be an attempt to do so. No more flagrant instance of the blindness or worse that is produced by intense prejudice could be adduced.

"Sub-section 4 is in effect merely an indirect method of making a woman liable to two years' imprisonment; for women constitute the vast majority of persons convicted of offences mentioned in the Schedule." Another wilful and flagrant violation of the truth. The offences mentioned in the Schedule are rape, indecent assault, exposure of the person or indecent exhibition, and loitering or importuning. Do women constitute the vast majority of persons convicted of rape? or of indecent assault? or of exposure of the person? Surely the hardihood of Dr. Wilson and her associates surpasses belief.

The declarants complain that the whole law on solicitation is in practice only applied to women. They might almost as well complain that the penalties for wife-beating are inflicted only on men. What would they have? If women constitute, as they do, the immense majority of the offenders, it appears to the mind unbiassed by sex-jealousy that they should constitute the majority of the convicted also.

At any rate, these manifestoes of Dr. Wilson and her associates will make sensible people very careful not to let women have any prominent part in the administration of the Act, when it becomes an Act, and not to listen very attentively to their complaints.—Ed. The Hospital.]

